# The Role of Human Papillomavirus Genotypes on Anal Squamous Intraepithelial Lesions Among Gay, Bisexual and Other Men Who Have Sex With Men Living With HIV: Beyond HPV‐16

**DOI:** 10.1002/jmv.70596

**Published:** 2025-09-09

**Authors:** Aroa Villoslada, Adrian Rodriguez, Patricia Sorni, Araceli Serrano, Andrea Salom, Carmen Collado, Mercedes García‐Gasalla, Antoni Payeras

**Affiliations:** ^1^ Hospital Universitari Son Llàtzer. Internal Medicine (Infectious diseases) Palma Spain; ^2^ Health Research, Institute of the Balearic Islands (IdISBa) Palma Spain; ^3^ Department of Medicine University of the Balearic Islands (UIB) Palma Spain; ^4^ Hospital Universitari Son Llàtzer. Microbiology Service Palma Spain; ^5^ Internal Medicine (Infectious diseases) Hospital Universitari Son Espases Palma Spain; ^6^ Centro de Investigación Biomédica en Red, Enfermedades Infecciosas (CIBERINFEC), Av Madrid Spain

**Keywords:** anal cancer, anal dysplasia, co‐testing, HPV, HSIL, PLHIV

## Abstract

Persistent high‐risk human papillomavirus (hHPV) infection, especially HPV‐16, plays a central role in the development of high‐grade squamous intraepithelial lesions (HSIL). This study aimed to evaluate the performance of co‐testing (cytology and hHPV detection) in a real‐world cohort of men who have sex with men (MSM) and transgender women (TW) living with HIV. We conducted a prospective study (2017–2023) at a tertiary care center in Spain. MSM and TW living with HIV underwent screening with anal cytology and PCR‐based hHPV testing. High‐resolution anoscopy (HRA) with biopsy was performed in cases with abnormal cytology and/or hHPV positivity. Clinical, epidemiological, and HIV‐related data were collected to identify risk factors for abnormal cytology and biopsy. 734 cytologies were performed in 380 participants. Abnormal cytology was found in 34%, most commonly ASCUS. hHPV was detected in 63.1% of samples; HPV‐16 was the most prevalent genotype (19.4%), present in 60% of HSIL cytologies and 65% of AIN2–3 biopsies. Risk factors for abnormal cytology included nadir CD4 < 200 cells/μL (aOR 2.61), prior condylomas (aOR 2.66), and infection with any oncogenic HPV genotype (aOR 4.12). Among 91 HRAs, 29.6% showed AIN2–3% and 2.1% in situ carcinoma. HPV‐16, HPV‐52, and HPV‐66 were most frequently associated with abnormal findings in the cytology. In conclusion, anal HPV infection was prevalent in MSM and TW living with HIV. The genotypes most frequently associated with abnormal cytology and histopathological findings were HPV‐16, HPV‐52, and HPV‐66. These findings highlight the potential value of implementing co‐testing strategies in anal dysplasia screening for this high‐risk population.

## 1 Introduction

Since the 1970s, the incidence of anal cancer has been increasing in the United States and Europe, accounting for 2.7% of all digestive cancers, with an overall incidence rate of 1.7 per 100,000 person‐years [1, 2, 3, 4, 5]. This risk is significantly higher in certain populations, particularly among people living with HIV (PLHIV), where it exceeds 60 per 100,000 person‐years. Within this group, men who have sex with men (MSM) are the most affected, with an anal cancer incidence ranging from 89 to 144 cases per 100,000 individuals [5, 6, 7, 8, 9].

Anal carcinoma is considered an opportunistic malignancy in PLHIV, primarily associated with persistent infection by high‐risk human papillomavirus (HPV), similarly to what has been observed in cervical cancer [10, 11]. The progression to cancer originates from precursor lesions such as high‐grade anal intraepithelial neoplasia (AIN II–III) or high‐grade squamous intraepithelial lesions (HSIL), which develop at the squamocolumnar junction of the anal canal [12, 13]. Persistent infection with high‐risk HPV (hHPV), particularly HPV‐16, has been identified as a key factor in the development of anal cancer, along with other risk factors such as sexually transmitted infections, a history of cervical cancer in cisgender women, dysplastic lesions, smoking, and older age [7, 14, 15, 16]. Although the progression of anal HSIL to invasive carcinoma is less common than in cervical HSIL, it has been well documented in PLHIV [17]. Screening programs for cervical HSIL have demonstrated efficacy leading to the implementation of anal HSIL screening strategies in PLHIV, though current recommendations remain heterogeneous [6, 18, 19, 20, 21, 22, 23]. The ANCHOR trial [24] provided strong evidence that HSIL treatment in PLHIV significantly reduces the risk of progression to anal cancer, reinforcing the need for structured screening programs.

However, diagnosing anal HSIL presents significant challenges. Cytology is the most widely used technique for detecting anal mucosal lesions [25]. Nonetheless, the sensitivity and specificity of cytology using the Papanicolaou test are highly variable, ranging from 47% to 93% and 32% to 50%, respectively. Sensitivity is higher in individuals with HIV and CD4 counts < 200 cells/μL, in those with high‐grade lesions, and in cases involving multiple affected anal quadrants. However, many studies report a low correlation between cytology findings and histopathological biopsy results [26, 27, 28]. Conversely, obtaining a biopsy requires high‐resolution anoscopy (HRA), a procedure that demands specialized training and is not always available in all healthcare settings. This underscores the need for additional biomarkers or risk stratification tools to optimize screening and focus efforts on high‐risk individuals.

The role of HPV testing in cervical cancer screening has gained prominence over cytology, particularly in women over 30 years old, as HPV prevalence in younger individuals is high (~ 80%), making it less effective as a primary screening tool [29]. However, there is ongoing debate regarding the utility of HPV testing in anal HSIL screening. Currently, there is no universal consensus regarding the use of HPV testing across international guidelines [21, 22, 30].

Since 2015, in our hospital, MSM living with HIV have undergone anal HSIL screening using cytology, followed by histological confirmation and ablative treatment when necessary. In June 2017, high‐risk HPV testing (14 genotypes) was incorporated into the screening protocol. Within this context, the present study aims to evaluate the outcomes of this screening strategy, implemented since June 2017, through co‐testing (cytology combined with hHPV detection) in a real‐world cohort of MSM living with HIV.

## 2 Methodology

### 2.1 Study Design and Population

We conducted a prospective study at Hospital Universitario Son Llàtzer, a tertiary care center in Palma de Mallorca, Spain, serving an estimated population of 300,000 inhabitants. The inclusion criteria were PLHIV, MSM, and TW, aged ≥ 18 years, who were being followed at the infectious diseases outpatient clinic and agreed to participate in the screening program for high‐grade anal dysplasia. Screening was offered to all eligible participants. All participants were asymptomatic at the time of screening.

**Figure 1:**
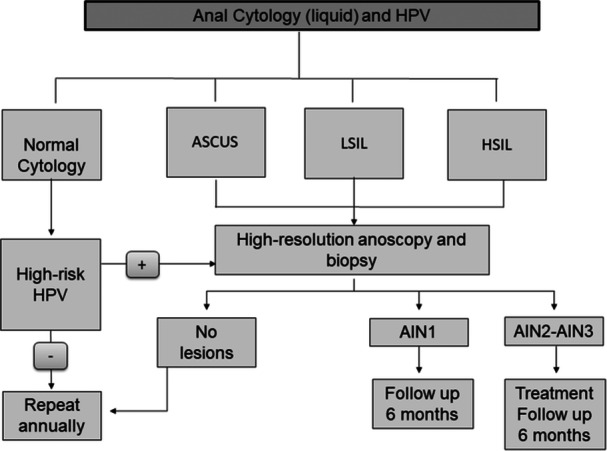
Schematic screening protocol.

In 2015, a high‐grade anal dysplasia detection program was initiated in our center. The initial assessment included an anal examination and cytology, followed by high‐resolution anoscopy (HRA) in patients with abnormal cytology results. Since June 2017, anal HPV testing has been incorporated into the protocol, with HRA conducted on patients who present with abnormal cytology and/or hHPV positivity, as outlined in Figure 1. The study period was from June 1, 2017, to December 31, 2023.

All participants were included in the Balearic eVIHa cohort, which comprises PLHIV in the Balearic Islands, Spain, whose research protocol was approved by the Research Ethics Committee of the Balearic Islands IB 3808/18 PI. All participants provided written inform consent before the inclusion in the cohort.

### 2.2 Sample Collection and Laboratory Procedures

During initial and follow‐up visits, two samples were collected using Dacron® swabs via anal smears: one for liquid‐based cytology (ThinPrep® solution), processed at the hospital's Pathology Department, and another sent to the Microbiology Department for hHPV detection by polymerase chain reaction (PCR).

Cytology samples were evaluated by a pathologist and classified according to the Bethesda System [31] as: (I) normal; (II) atypical squamous cells of undetermined significance (ASCUS); (III) low‐grade squamous intraepithelial lesion (LSIL); (IV) high‐grade squamous intraepithelial lesion (HSIL); (V) anal squamous cell carcinoma (SCC).

Samples analyzed in the Microbiology Department were tested for hHPV using the Anyplex HPV14 reagent from Seegene® (Werfe) processed using a highly multiplexed real‐time PCR automated system [32], which detects the following oncogenic genotypes: HPV‐16, HPV‐18, HPV‐31, HPV‐33, HPV‐35, HPV‐39, HPV‐45, HPV‐51, HPV‐52, HPV‐56, HPV‐58, HPV‐59, HPV‐66, and HPV‐68.

All samples were collected at the hospital by trained healthcare professionals (nurses or physicians) and were not self‐administered.

### 2.3 High‐Resolution Anoscopy (HRA)

HRA was performed by infectious disease physicians trained in the procedure, following standard guidelines [33]. Acetic acid and Lugol's iodine were applied to identify potential squamous intraepithelial lesions (SILs), which were then biopsied. No random biopsies were performed. Patients diagnosed with AIN2–AIN3 (high‐grade anal intraepithelial neoplasia) or SCC were referred to the proctology unit of the general surgery department of the same hospital for surgical ablation [25] and continued follow‐up within the anal dysplasia screening program (Figure 1). In cases where more than one biopsy was taken during the same procedure, the most severe histological result was considered for analysis.

### 2.4 Other Variables Collected

Clinical, epidemiological, and laboratory data were extracted from the electronic medical records system for HIV care in the Balearic Islands (eVIHa).

Epidemiological and clinical variables include age, smoking status, HIV stage (Centers for Disease Control and Prevention, CDC), duration of antiretroviral therapy (ART) from baseline, time since HIV diagnosis, history of anal or genital condylomas, and history of SCC. Laboratory variables were those related to HIV infection: nadir CD4, current CD4 count, and viral load at study inclusion.

### 2.5 Statistical Analysis

Frequency distributions (number and percentages) and measures of central tendency (mean and standard deviation) were used to describe the study population and the main variables.

We compared clinical and demographic variables between independent groups defined by cytology results (benign vs. LSIL/HSIL) and biopsy results (AIN2/3 vs. benign cytology) using the Chi‐squared test (*χ*²) or Fisher's exact test for categorical variables. No correction for multiple comparisons was applied due to the limited number of tests performed. Cytology samples deemed unsatisfactory due to insufficient material were excluded from the analysis.

Additionally, we performed univariate and multivariate logistic regression analyses to explore risk factors for LSIL/HSIL, including age, years since HIV diagnosis, nadir CD4 < 200 cells/µL, current smoking status, history of neoplasia, history of condyloma, and infection with at least one hHPV genotype.

The prevalence of different hHPV genotypes was calculated as absolute numbers and percentages of the total samples, excluding those with unsatisfactory cytological results. We also examined the relationship between the prevalence of each hHPV genotype and cytology and biopsy results.

A *p*‐value < 0.05 was considered statistically significant. No correction for multiple comparisons was applied due to the limited number of tests performed. Statistical analyses were conducted using Python in Google Colab.

## 3 Results

### 3.1 Epidemiological and Clinical Characteristics of the Cohort

**Figure 2:**
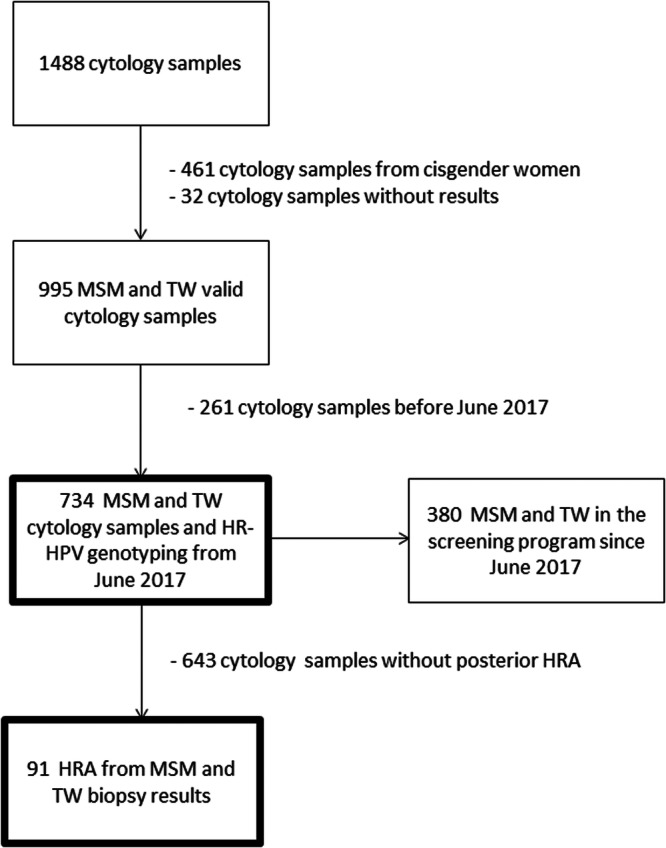
Flowchart of the study.

A total of 1488 anal cytology swabs have been performed in our hospital since 2015. From them, 734 cytology tests were performed on 380 MSM and TW living with HIV since June 2017 (Figure 2), with a median of 2 cytology swabs per patient. The mean age at first cytology was 44 years (SD 11). During screening, 21% (*n* = 79) of participants were under 35 years old. The average time since HIV diagnosis was 14 years (SD 8), and they had been on ART 11 years (SD 7). The mean nadir CD4 count was 493 cells/μL (SD 302), with 90.3% (*n* = 343) of patients having viral loads < 50 copies/mL at screening inclusion (Table 1).

**Table 1:** Baseline characteristics from the 380 MSM and TW in the screening program since June 2017. Results are expressed as *N* (%) unless otherwise specified.

Characteristic	*N* (%) or mean (SD)
MSM	373 (98.2%)
TW	7 (1.8%)
Age (years), mean (SD)	44 (11)
HIV RNA VL < 50 copies/mL	343 (90.3%)
Nadir CD4 T‐cell count < 200 cel/µL	60 (15.8%)
CDC C3 category	35 (9.4%)
INSTI ART based	283 (74.5%)
PI ART based	30 (7.9%)
NNRTI ART based	67 (17.6%)
Smoking status	100 (26.3%)
HPV vaccination	21 (5.5%)
Previous neoplasia	41 (10.8%)
Previous condylomas	59 (15.5%)

Abbreviations: ART, antiretroviral treatment; HPV, human papillomavirus; INSTI, integrase strand transfer inhibitor; MSM, men who have sex with men; NNRTI, non‐nucleoside reverse transcriptase inhibitors; PI, protease inhibitors; TW, transgender women; VL, viral load.

### 3.2 Dysplastic Lesions Detected by Cytology and Biopsy

During the study period, 34% (*n* = 129) of MSM living with HIV had abnormal cytology results (ASCUS, LSIL, or HSIL). The most frequent finding was ASCUS (*n* = 85, 12.1%), followed by LSIL (*n* = 47, 6.4%), and HSIL (*n* = 10, 1.4%). A total of 85 cytology samples (11.5%) were non‐evaluable (unsatisfactory due to the absence of epithelial cells in the swab).

When comparing clinical characteristics between patients with LSIL/HSIL versus normal cytology, those with abnormal cytological results were more likely to be smokers, had a longer duration of HIV infection, had a higher prevalence of nadir CD4 < 200 cells/μL, a greater history of malignancies, and a history of condylomas (Table 2). Additionally, abnormal cytology was associated with a higher prevalence of at least one oncogenic HPV genotype (Table 2). In the multivariate analysis, CD4 nadir < 200 cells/μL, a history of condylomas, and the presence of any oncogenic HPV genotype were the risk factors that remained independently associated with having abnormal cytologies (Table 2).

**Table 2:** Comparison of the potential predictors of anal dysplasia between benign vs LSIL/HSIL cytology.

	Benign cytology (*N* = 502)	LSIL/HSIL cytology (*N* = 57)	Univariate OR (95% CI)	*p* value	Multivariate OR (95% CI)	*p* value
Age (years), mean (SD)	46 (11)	46 (13)	0.99 (0.97–1.02)	0.76	0.96 (0.93–1.00)	0.04
Years since HIV diagnosis, mean (SD)	15 (8)	17 (9)	1.03 (1.00–1.06)	0.06	1.04 (1.00–1.09)	0.07
Nadir CD4 T‐cell count < 200 cel/µL	74 (14.7%)	17 (29.8%)	2.42 (1.30–4.49)	0.005	2.61 (1.30–5.27)	0.007
Current smoking status	138 (27.5%)	23(40.4%)	1.75 (1.00–3.08)	0.05	1.68 (0.92–3.09)	0.09
Previous neoplasia	49 (9.8%)	13 (22.8%)	2.69 (1.36–5.34)	0.005	1.60 (0.68–3.77)	0.28
Previous condylomas	69 (13.7%)	21 (36.8%)	3.60 (1.99–6.53)	< 0.0001	2.66 (1.39–5.07)	0.003
With at least one HPV genotype	298 (59.4%)	49 (86.0%)	4.12 (1.91– 8.88)	< 0.001	4.45 (2.00–9.88)	< 0.001

Abbreviations: HPV, human papillomavirus; HSIL, high‐grade squamous intraepithelial lesion; LSIL, low‐grade squamous intraepithelial lesion; SD, standard deviation.

A total of 91 HRAs with multiple biopsies were performed, revealing high‐grade dysplasia (AIN2–AIN3) in 29.6% (*n* = 27), low‐grade dysplasia (AIN1) in 33% (*n* = 30), and in situ carcinoma in 2.1% (*n* = 2).

When comparing patients with AIN2–AIN3 biopsy results to those with normal cytology, individuals with high‐grade lesions had a higher prevalence of nadir CD4 < 200 cells/μL (41.4% vs. 14.7%; *p* < 0.001) a greater history of condylomas (34.5% vs. 13.7%; *p* = 0.005), and a prior history of malignancies (27.6% vs. 9.8%; *p* = 0.007). Moreover, the percentage of participants without any HPV genotype was significantly lower in those with AIN2‐AIN3 lesions than in those with normal cytology (6.7% vs. 40.6%, *p* < 0.001).

Table 3 shows the concordance of cytology and biopsy results. As can be seen, participants with HSIL on cytology were much more likely to have AIN2‐AIN3 on biopsy, whereas those with ASCUS were more frequently found to have AIN1 or no dysplasia.

**Table 3:** Concordance between the results of anal cytology and anal biopsy. Results are expressed as *N* (%) of the total cytological results.

Biopsy results	Cytology results			
	Benign (*N* = 10)	ASCUS (*N* = 46)	LSIL (*N* = 26)	HSIL (*N* = 8)
Without dysplasia	4 (40%)	15 (32.6%)	12 (46.2%)	1 (12.5%)
AIN1	2 (20%)	20 (43.5%)	6 (23.1%)	1 (12.5%)
AIN2 or worse	4 (40%)	11 (23.9%)	8 (30.7%)	6 (75%)

Abbreviations: AIN, anal intraepithelial neoplasia; ASCUS, atypical squamous cells of undetermined significance; HSIL, high‐grade squamous intraepithelial lesion; LSIL, low‐grade squamous intraepithelial lesion.

### 3.3 High‐Risk HPV Detection

The prevalence of the different hHPV genotypes are shown in Table 4. Overall, 63.1% (*n* = 411) of samples tested positive for at least one hHPV genotype. Among these, 59.4% (*n* = 244) had ≥ 2 genotypes, and 19% (*n* = 77) had ≥ 4 genotypes.

**Table 4:** Prevalence of the different studied HR‐HPV genotypes. Results are expressed as *N* (%).

	Prevalence
HPV‐16	136 (21.0%)
HPV‐18	49 (7.6%)
HPV‐31	72 (11.1%)
HPV‐33	63 (9.7%)
HPV‐35	44 (6.8%)
HPV‐39	70 (10.8%)
HPV‐45	45 (6.9%)
HPV‐51	49 (7.6%)
HPV‐52	93 (14.3%)
HPV‐56	36 (5.5%)
HPV‐58	68 (10.5%)
HPV‐59	51 (7.8%)
HPV‐66	72 (11.1%)
HPV‐68	81 (12.5%)

*Note:* HR‐HPV samples when cytological results were unsatisfactory were excluded.

The most prevalent oncogenic HPV genotypes were HPV‐16 (19.4%), followed by HPV‐52 (13.4%), and HPV‐68 (12.2%) (Table 4).

**Figure 3:**
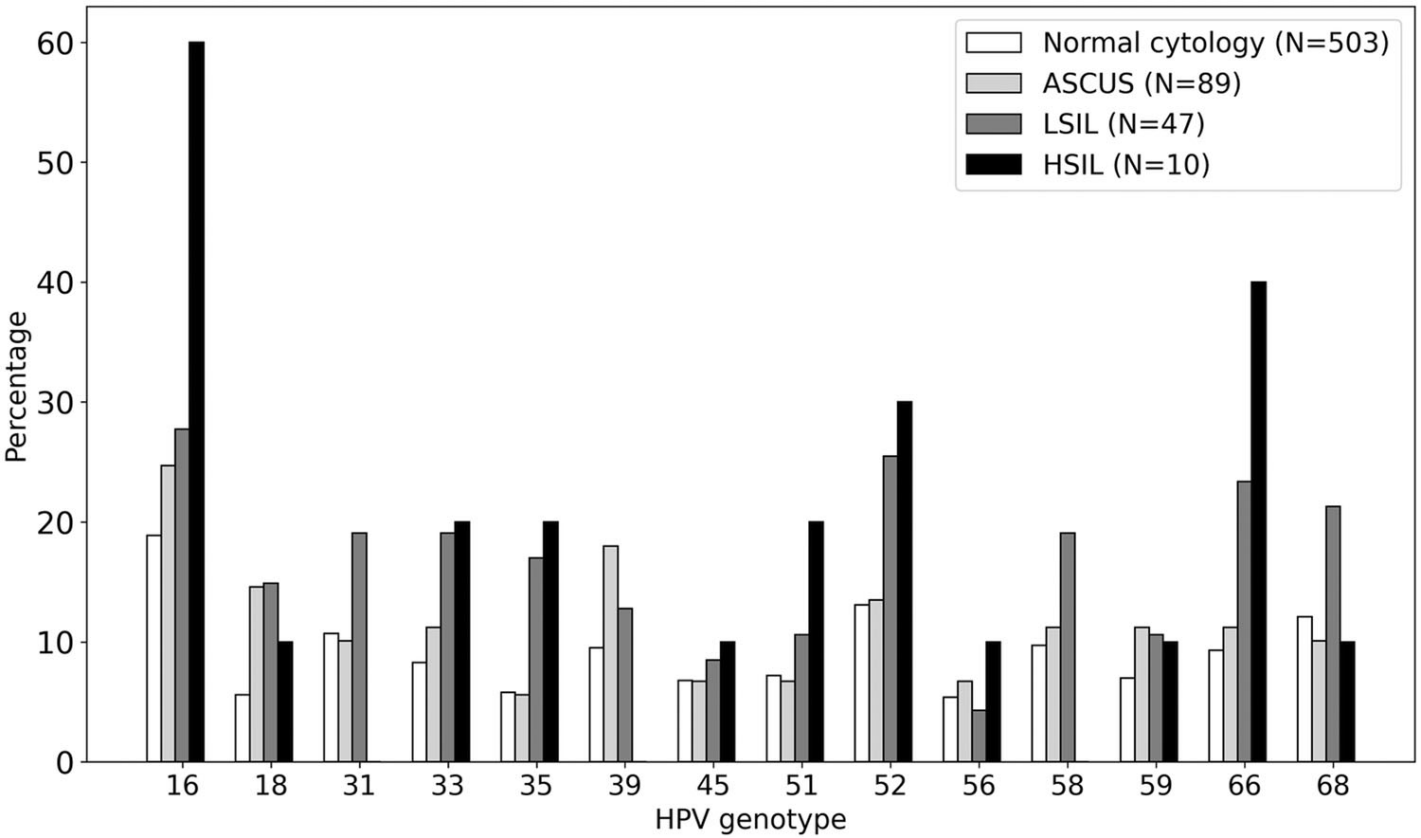
Percentage of HPV genotype positivity across cytological classifications (Normal, ASCUS, LSIL, HSIL).

Figure 3 shows the relationship between cytological results and the positivity of each hHPV genotype. In general, all genotypes were numerically more prevalent when any abnormality was present in the cytology. The hHPV genotypes more associated with abnormal cytology were HPV‐16, HPV‐66, and HPV‐52. In fact, HPV‐16 was present in 60% of HSIL cytology samples.

**Figure 4:**
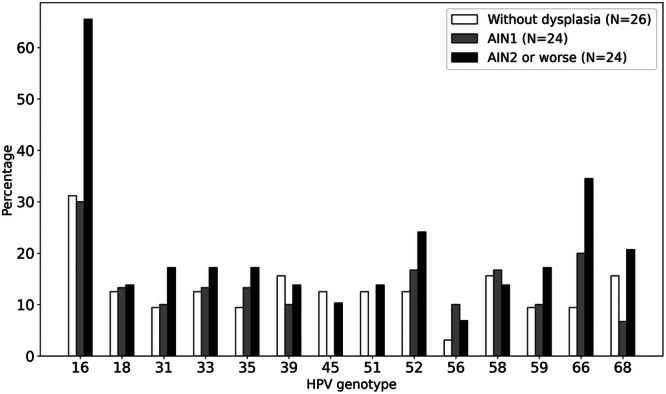
Percentage of HPV genotype positivity across biopsy result groups (Without dysplasia; AIN1 or AIN2 or worse).

Similar results are obtained when examining the relationship between anal biopsies and hHPV genotypes (Figure 4). Again, the hHPV genotypes more associated with abnormal biopsy were HPV‐16, HPV‐66, and HPV‐52. HPV‐16 was present in 65% of AIN2–AIN3 biopsies.

## 4 Discussion

Our study provides a real‐world perspective on the longitudinal follow‐up of a cohort of MSM and TW living with HIV, screened for high‐grade anal dysplasia over 7 years using a co‐testing approach that includes cytology and hHPV genotyping. Our results indicate that, in addition to HPV‐16, other genotypes such as HPV‐52 and HPV‐66 are also associated with abnormal results in both cytology and biopsy.

We found a high prevalence of hHPV in this population, a finding consistent with previous studies [34, 35]. More than 60% of the analyzed samples tested positive for hHPV, with nearly 20% coinfected by more than four genotypes. Notably, HPV‐16 was detected in 20% of cases and was strongly associated with HSIL, being present in 60% of such cases. These findings reinforce the necessity of continued screening in this high‐risk population, given its vulnerability to HPV‐related malignancies, including anal cancer.

A comparable study conducted in northern Spain by Pérez‐González et al. [36] identified hHPV in nearly 80% of cases, with HPV‐16 present in 23%. Similarly, Hernández et al. [37] reported an HPV‐16 prevalence of 19% in a cohort of MSM with HIV over 50 years old who had HSIL‐compatible cytology. Despite the lower prevalence of that genotype, HPV‐16 remained a significant risk factor for HSIL. One aspect not assessed as in our study or in the Hernández is the persistence of hHPV and its relationship with HSIL development. Persistent HPV infection may explain differences in HPV‐16 prevalence across cohorts, with those unable to clear the infection being at increased risk of HSIL. Larger cohorts may exhibit lower overall HPV infection rates, but the persistence of infection may contribute to a higher HSIL incidence. More research is needed on HPV persistence and its role in the progression of anal dysplasia.

Our data also underscore the utility of HPV‐16 identification in stratifying patients at higher risk for HSIL, aiding in the selection of candidates for high‐resolution anoscopy (HRA). Interestingly, HPV‐18 did not show a significant presence in HSIL cytology, a finding consistent with previous research [35], where HPV‐16 was detected in 50% of HSIL cases compared to 22% for HPV‐18. Some authors [38, 39] suggest that HPV‐16 has a higher oncogenic potential due to its persistence, making it more difficult for the immune system to clear. A meta‐analysis by Megan A. Clarke et al. [20] highlighted the potential benefit of co‐testing in anal HSIL screening, showing that HPV‐16 testing had a sensitivity of 43% and specificity of 83%, whereas HPV‐18 testing did not enhance detection rates.

Other HPV genotypes associated with abnormal cytological results in our cohort were HPV‐52 and HPV‐66, being the last one classified as a “probable” high‐risk genotype [40]. Limited data exist on the significance of these genotypes in anal HSIL screening among MSM with HIV. Kamwing Jair et al. [41] reported an association between HPV‐33, HPV‐35, and HPV‐56 with HSIL compared to LSIL, while Chowdhury et al. [42] identified HPV‐35/51 as potential HSIL risk factors. The heterogeneity of study populations and differences in analyzed genotypes complicate direct comparisons between studies.

Nonetheless, it has been suggested that multiple HPV infections may correlate with higher HSIL rates, possibly serving as a marker for persistent disease and progression [42].

Given the emerging evidence on the role of hHPV detection in HSIL screening among MSM with HIV, the 2024 American Society of Anal Cancer guidelines [18] have incorporated hHPV testing into anal dysplasia screening protocols.

In recent years, HPV vaccination has demonstrated efficacy in reducing HPV‐related cancer rates. A clinical trial by Goldstone et al. [43] showed a significantly lower anal cancer incidence among recipients of the quadrivalent HPV vaccine (20.5 vs. 906.2 cases per 10,000 person‐years). However, HPV vaccination rates in our cohort were too low to draw meaningful conclusions. Since 2023, the Spanish Ministry of Health has funded nonavalent HPV vaccination for MSM under 45 years of age [44], a measure that may substantially impact HPV‐related cancer prevention. Nevertheless, HPV‐66, which was prevalent in our cohort, is not covered by the current vaccine. Additionally, mass vaccination in the female population may be altering HPV genotype distribution, potentially leading to the emergence of previously low‐risk genotypes in anal dysplasias.

Our cohort exhibited a high prevalence of dysplastic lesions, with ASCUS being the most frequent cytological finding and HSIL representing only 1.4%. Non‐evaluable cytology accounted for 11.5% of cases, likely reflecting the initial learning curve in screening procedures among clinicians and pathologists. Variability in screening methodologies and studied populations complicates direct comparisons with other series. In the Pérez‐González et al. cohort [36], HSIL prevalence was 3.9%, compared to 33% reported by Sanger et al. [45] and 5.9% by Wells et al. [46]. Notably, Wells et al. emphasized that stigma and poor health status reduced patient adherence to HRA.

We performed 91 HRAs, diagnosing two cases of carcinoma in situ and 27 cases of AIN2/AIN3, representing 30% of biopsied lesions. This HSIL detection rate is higher than that reported in the Pérez‐González et al series [36]. Of particular interest, 24% of ASCUS‐compatible cytology cases subjected to HRA revealed HSIL (AIN2/AIN3). Moreover, nearly 25% of ASCUS cases harbored HPV‐16. Among normal cytology cases that underwent HRA, 40% showed AIN2/AIN3, prompting reconsideration of screening approaches, as HPV‐16 and other high‐risk HPV genotypes guided the decision to perform HRA. These findings support the role of hHPV genotyping in optimizing anal HSIL screening strategies.

Several factors correlated with HSIL in our cohort, including HIV infection duration, severe immunosuppression (nadir CD4 < 200), history of malignancies, and prior condylomas, findings consistent with previous studies [47, 48]. Salgado et al. [49] attempted to develop a predictive model for HSIL risk, suggesting that individual patient characteristics, in addition to cytology results, could aid in prioritizing HRA referrals. These data are in concordance with findings from Llibre et al. [50], who analyzed anal cancer cases in the Catalan‐Balear cohort.

This real‐world descriptive study on anal dysplasia screening using co‐testing has several limitations. First, as in many healthcare settings, HRA availability was limited. As a result, clinicians often prioritized HRA for patients considered at higher risk based on factors such as age, smoking status, and comorbidities. Second, participant adherence to HRA was suboptimal, although specific data on this aspect were not collected. Limited adherence affects the evaluation of screening's impact and effectiveness. Third, due to the small number of participants with AIN2–AIN3 lesions, we were unable to perform a multivariate analysis as we did for abnormal cytology results. Fourth, comparison between MSM and TW was not possible due to the limited number of TW in this cohort. Furthermore, we analyzed 14 high‐risk HPV genotypes, but other genotypes, including low‐risk types, may also be relevant and were not assessed in this study. Lastly, we did not collect data on patients’ sexual practices to ascertain a more comprehensive assessment of behavioral risk factors associated with anal dysplasia.

## 5 Conclusions

HPV infection is highly prevalent among MSM and TW living with HIV. In our cohort, anal dysplastic lesions were common, with ASCUS being the most frequently detected cytological abnormality. An association between HPV‐16, HPV‐52, and HPV‐66 and HSIL was observed, highlighting the potential utility of co‐testing in anal dysplasia screening for this high‐risk population. Our findings support the integration of hHPV genotyping into screening strategies to improve the detection and management of HSIL, ultimately enhancing outcomes for individuals living with HIV.

## Author Contributions

**Aroa Villoslada:** conception and design of the study, sample collection, manuscript preparation. **Adrian Rodriguez:** conception and design of the study, data analysis, manuscript preparation. **Patricia Sorni:** conception and design of the study, sample collection, manuscript review. **Araceli Serrano:** conception and design of the study, sample collection, manuscript review. **Andrea Salom:** conception and design of the study, sample collection, manuscript review. **Carmen Collado:** conception and design of the study, sample analysis, manuscript review. **Mercedes García‐Gasalla:** conception and design of the study, data analysis, manuscript review. **Antoni Payeras:** conception and design of the study, data analysis, manuscript review.

## Conflicts of Interest

A.V. and P.S. have received speaker fees from Gilead Sciences, Janssen, and ViiV, outside of the submitted work. A.S., A.S., C.C., A.P., M.G.G., and A.R. have no conflicts of interest.

## Data Availability

The data that support the findings of this study are available from the corresponding author upon reasonable request. The data that support the findings of this study, as well as the code, are available from the corresponding author upon reasonable request.
